# Safety of Repeated Administration of Parenteral Ketamine for Depression

**DOI:** 10.3390/ph13070151

**Published:** 2020-07-13

**Authors:** David Feifel, David Dadiomov, Kelly C. Lee

**Affiliations:** 1 Department of Psychiatry, University of California, San Diego, CA 92037, USA; 2 Kadima Neuropsychiatry Institute, San Diego, CA 92037, USA; 3 Department of Clinical Pharmacy, University of Southern California, Los Angeles, CA 90089, USA; dadiomov@usc.edu; 4 Skaggs School of Pharmacy and Pharmaceutical Sciences, University of California San Diego, La Jolla, CA 92037, USA; kellylee@health.ucsd.edu

**Keywords:** ketamine, major depressive disorder, depression, addiction

## Abstract

The objective of this study was to investigate the safety of repeated parenteral ketamine for depression. An electronic survey inquiring about the frequency of adverse events was distributed to providers of parenteral ketamine for depression. In addition, the investigators conducted a search of published studies describing six or more repeated parenteral ketamine treatments administered to individuals for depression, and extracted reported adverse events. The survey was sent to 69 providers, of which 36 responded (52% response rate); after eliminating those that were incomplete, 27 were included in the analysis. The providers in the analysis collectively reported treating 6630 patients with parenteral ketamine for depression, one-third of whom received more than 10 treatments. Only 0.7% of patients experienced an adverse effect that required discontinuation of ketamine. Psychological distress during the treatment was the most frequent cause. Other adverse events were extremely rare (such as bladder dysfunction (0.1%), cognitive decline (0.03%) and psychotic symptoms (0.03%)). Among the 20 published reports of repeated parenteral ketamine treatments, rates of significant adverse events resulting in discontinuation were low (1.2%). The rate of adverse effects reported in the survey and the published literature is low, and suggests that long-term treatment of depression with ketamine is reasonably safe.

## 1. Introduction

Depression is one of the leading causes of disability worldwide, with an estimated 322 million people living with depression [1,2]. The mainstay of treatment at present is antidepressant medications; however, it is estimated that at least half the patients treated with conventional antidepressants do not experience remission despite multiple trials of different agents [3]. Since traditional antidepressants typically take weeks to produce therapeutic effects, and the likelihood of therapeutic effects diminish with each failed trial of conventional antidepressants [3], several failed trials of antidepressants may subject patients to prolonged depressive periods. The diminishing chances of success with each trial of an antidepressant is likely due, in part, to their highly circumscribed, and relatively similar, mechanisms of actions. All traditional antidepressants regulate synaptic transmission of monoamines, which only address one of the many purported pathologies of depression [4]. This underscores the need for antidepressant treatments that utilize novel mechanisms of action.

One such novel treatment that has gained significant attention is ketamine. Ketamine was developed as an anesthetic, and is FDA-approved for this indication [5]. For more than a decade, evidence has been growing that, at subanesthetic doses, ketamine produces a reduction in depressive symptoms in patients with treatment resistant depression (TRD) [6]. The antidepressant effect is often seen within 24 h of treatment, and persists days or weeks beyond treatment. Recently, intranasal s-ketamine, an enantiomer of racemic ketamine, was FDA-approved for the treatment of TRD in conjunction with a conventional antidepressant [7]. Despite ketamine’s robust antidepressant effect, repeated administrations, typically at one- to four-week intervals, are required to maintain the therapeutic benefits [6].

There is concern that long-term repeated ketamine administrations may produce adverse effects [8]. The most cited concerns are addiction [9], damage to the urinary tract [10], psychosis [11] and cognitive decline [12]. These concerns are generally extrapolations of the adverse effects observed occasionally with illicit use of ketamine. However, several factors limit the generalizability of the illicit use of ketamine to its therapeutic counterpart. For example, ketamine used illicitly may contain adulterants, may be self-administered concurrently with other illicit substances, or may be used at higher doses [13]. In contrast, ketamine treatment for depression is most commonly administered parenterally under the supervision of a physician, and patients are typically monitored while they experience the characteristic dissociative experience that usually lasts 40‒60 min.

A 2018 meta-analysis summarized the side effects observed in past ketamine trials, and noted that there was a general dearth of serious adverse effects among those trials. However, the authors also noted that most studies published to date involve single or small number infusions in subjects, and there is thus a dearth of data regarding long-term outcomes [14]. Despite this dearth of evidence concerning the long-term use of ketamine for depression, a growing number of providers have begun to use ketamine to treat refractory depression, often in clinics that focus on offering this treatment [15]. Thus, there is a strong need to assess the relative safety of repeated ketamine administration, to induce and potentially maintain the drugs acute antidepressant efficacy.

In the absence of long-term prospective studies or data derived from an established database or registry, provider surveys are often utilized as a method of obtaining information about the safety of treatments [16,17,18]. The objective of this study was to investigate the safety of repeated parenteral ketamine administration for depression in ‘real world’ clinical settings, using a survey targeting experienced providers of ketamine. While ketamine is used to treat depression via several routes of administration, including intranasal (IN) and oral (PO), we limited our survey to the parenteral routes, namely intravenous (IV) and intramuscular (IM) routes. Intravenous ketamine was the route used in the original clinical trials demonstrating the drug’s antidepressant efficacy, and most subsequent studies have reported using this route. It has also been reported to be the most common route utilized by community providers [6]. Furthermore, in contrast to parenteral ketamine, which is overwhelmingly administered and monitored in medical office or inpatient settings, IN and PO ketamine are frequently prescribed for self-administration by the patient without direct supervision. We excluded these formulations due to the uncertainty of doses and the inability to accurately capture the extent and nature of the adverse effects.

In conjunction with the survey, we also conducted a literature review of published papers reporting multiple (>6) parenteral administrations of ketamine in patients with depression, in order to extract information on the safety and tolerability of this treatment practice and compare it with the results of our survey.

## 2. Results

### 2.1. Survey

The electronic survey was distributed to 69 providers of parenteral ketamine for depression. Responses were obtained from 36 participants (52% response rate). Eight surveys were excluded from analysis as they did not complete the questions about the numbers of adverse effects of ketamine. One response was excluded from analysis due to a high degree of internal inconsistency. Therefore, 27 survey responses were included in the final analysis.

The medical specialty and nature of the practice site of the ketamine providers, as well as the number of patients they have treated with parenteral ketamine, are provided in Table 1.

**Figure 1:**
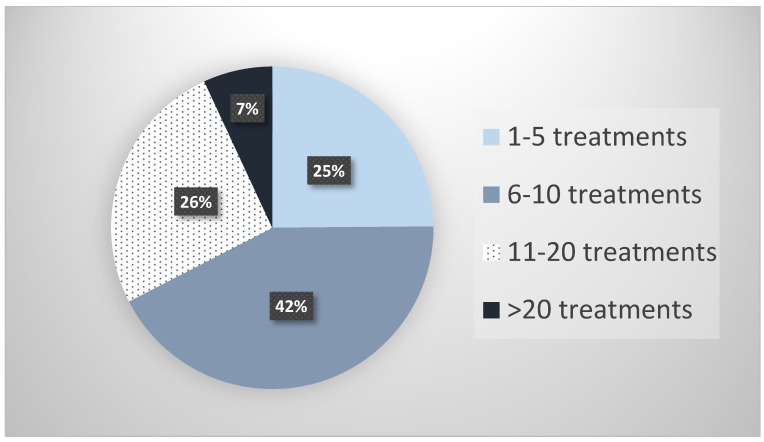
Number of parenteral ketamine treatments received by patients as reported by providers.

Collectively, the 27 survey respondents reported treating 6630 patients with parenteral ketamine for the treatment of depression. The median number of patients treated was 80. Figure 1 illustrates the breakdown of these 6630 patients in terms of the number of parenteral ketamine treatments they received. Approximately a third of these patients (34%) had been under treatment with parenteral ketamine for longer than 12 months, and a third (33%) had received more than 10 individual ketamine treatments.

After reviewing all reported adverse events and eliminating any that did not meet the stated definitions for inclusion (e.g., parenteral route, TRD indication, etc.), there were 47 patients out of the 6630 patients treated (0.7%) who required discontinuation due to an adverse effect during the treatment of depression with parenteral ketamine (Table 2).

The most commonly reported adverse effect that required discontinuation was psychological distress during treatment, which occurred in 33 patients (0.5%). Other adverse events that prompted discontinuation included nausea (*n* = 6), bladder dysfunction (*n* = 3), mania/hypomania (*n* = 2) and psychotic symptoms (*n* = 1). Spontaneously reported adverse events from providers included respiratory distress (*n* = 1) and seizure (*n* = 1).

### 2.2. Literature Review

Our literature search revealed 19 published papers that met our stated criteria (reporting on the effects of six or more repeated ketamine treatments administered parenterally to people with TRD). Table 3 summarizes the adverse events reported in these 19 published studies.

aan het Rot et al. [19] administered six ketamine infusions (0.5 mg/kg over 40 min), over 12 days, to 10 medication-free TRD patients with continuous vital sign monitoring. Aside from the expected significant dissociative effects in some patients, no significant adverse events were observed.

Murrough et al. [20] reported administering six ketamine infusions (0.5 mg/kg over 40 min), over 12 days, to 21 TRD patients. None of these patients experienced any serious side effects during the infusion series or during the follow-up period that extended up to 83 days. Zhou et al. [21] administered six ketamine infusions (0.5 mg/kg over 40 min), over 12 days, to 84 patients with unipolar and bipolar depression. Severity of depressive symptoms and several domains of neurocognition were assessed at baseline, one day following the last infusion, and again two weeks post-infusion. They reported that one patient withdrew due to a manic switch, but there were no other notable adverse events. As for neurocognition, they found significant improvements in speed of processing and verbal learning, and no deterioration in the other cognitive domains. Archer et al. [22] conducted a chart review identifying 11 patients who received between 10 and 51 ketamine infusions (0.5 mg/kg over 40 min) over a period 6 to 49 weeks. They found no serious adverse events. Wilkinson et al. [23] described 14 patients who received 12‒45 ketamine infusions (0.5 mg/kg over 40 min) over 14‒126 weeks. Aside from a small number of patients (*n* = 4) who had a transient increase in BP during some of their infusions, they witnessed no significant adverse events, and concluded that “Ketamine treatment was generally well tolerated in patients who received infusions on a long-term basis.” Phillips et al. [24] conducted a study of 41 subjects with treatment-resistant depression, who received single blinded infusions of ketamine and midazolam, followed by six open-label ketamine infusions administered thrice weekly for two weeks, followed by another four weekly infusions. Participants experienced transient elevations of blood pressure and heart rate, but the authors reported no serious adverse events, and no evidence of craving or drug-seeking behavior during the infusions or follow-up. None of the participants had to discontinue infusions due to cardiorespiratory effects, and no rescue medications were necessary during the study.

Vande Voort et al. [25] administered six IV ketamine infusion (0.5 mg/kg over 40 min), thrice weekly over two weeks, to subjects with TRD. Five of the subjects received four additional weekly infusions, and were followed for 4 weeks after the last infusion. They observed only mild and transient adverse events; however, one subject developed behavioral outbursts and suicide threats during the 4-week post treatment follow-up period, and another patient died by suicide several weeks after the follow-up period. The treatment providers did not believe these events were related to the previous ketamine treatments.

Ionescu et al. [26] conducted a study of 14 MDD patients with persistent suicidal thoughts. They received six weekly ketamine infusions, the first three at 0.5 mg/kg over 40 min, followed by three at 75 mg/kg over 40 min. They measured dissociation using the Clinician-Administered Dissociative States Scale (CADSS), but did not specifically report these results, nor did they report adverse effects.

Another study by Ionescu et al. [27] reported 26 patients with TRD and chronic current suicidal ideation, who were randomized in a double-blind manner to receive six ketamine infusions (0.5 mg/kg over 45 min) or saline over 3 weeks. Predictably, patients who received ketamine reported significantly higher dissociative symptoms (*p* < 0.001) on the CADSS during the infusion visits, with the largest difference shown at 30 min after the start of the infusion (*p* < 0.01). No other adverse effects were reported.

Singh et al. [28] conducted a study in which adults with TRD received IV ketamine infusions (0.5 mg/kg over 40 min) either thrice weekly (6‒12 infusions, *N* = 11) or twice weekly (8 infusions, *N* = 10). The authors report that both regimens were generally well tolerated

Mild emergent adverse events during the infusions included headache, anxiety, dissociation, nausea and dizziness, which were observed in 16‒28% of twice-weekly group, and 6‒41% of the thrice-weekly group, and all were resolved within 3 h after the infusion. Two patients discontinued due to ketamine-related emergent adverse effects in the twice-weekly group; one due to increased anxiety and another due to increased anxiety and paranoia. In the thrice-weekly group, one patient discontinued due to ketamine-related symptoms, reported as anxiety, dizziness, hypoesthesia and feeling cold.

Shiroma et al. [29] conducted an open-label study of six ketamine infusions (0.5 mg/kg over 40 min) administered over 12 days in 12 TRD patients. The infusions were well tolerated, aside from a single episode of transient increase in blood pressure (180/92) and another single episode of vomiting, effectively countered by IV labetalol and ondansetron, respectively.

Diamond et al. [30] enrolled 28 unipolar and bipolar TRD patients to receive a series of either three weekly (*n* = 15) or six twice-weekly (*n* = 13) infusions of ketamine (0.5 mg/kg over 40 min).Two patients discontinued before receiving all their infusions because of adverse reactions attributable to ketamine; one for a panic attack experienced during the infusion and the other for a vasovagal episode experienced during the fourth infusion. Other adverse events reported that did not result in discontinuation included panic attack (*n* = 1), rapid cycling of mood with a mild hypomanic episode (*n* = 1), emesis (*n* = 2) during treatment, symptomatic cystitis (*n* = 1) and a hypnogogic hallucination (*n* = 1).

Bryant et al. [31] reported on six geriatric patients (65‒82 years old) who received 8‒22 ketamine IV infusions (0.5 mg/kg over 40 min) every 2‒6 weeks, based on individual maintenance interval needs. They noted only mild, transient adverse effect.

George et al. [32] reported on 10 geriatric subjects (≥60 years) with MDD or bipolar disorder, who received 12 open-label subcutaneous ketamine injections (0.3‒0.5 mg/kg) administered twice per week for 1 month, and then weekly for one month. The injections were administered 6 months after patients received a series of three to five subcutaneous ketamine injections during a dose ranging phase. The investigators reported mild perceptual disturbances (colors or sounds seemed different), derealization, altered body perception and altered time perception, with peak effects occurring 10‒15 min after injection, resolving without intervention by 40 min after injection in all sessions. No significant treatment-emergent adverse psychiatric symptoms were reported at any time point. There were small transient increases in systolic and diastolic blood pressure, peaking at 4 h after ketamine, and four instances of heart rate increases that exceeded 120% of baseline, which never exceeded 131.5. Five patients reported mild neurologic symptoms (dizziness, *n* = 5; numbness, *n* = 2, headache, *n* = 1) with full resolution within 2 h. One patient reported the urge to urinate slightly more often. The liver function tests as well as neuropsychological test scores were all within normal limits, except in one patient whose aspartate aminotransferase (42‒60) and alanine aminotransferase (25‒44) were elevated after 12 treatments of 0.5 mg/kg.

Albott et al. [33] reported an open label study of 15 patients with TRD, who received a series of six ketamine infusions (0.5 mg/kg over 40 min). The authors reported no serious adverse events and no emergent adverse events, other than a transient increase in dissociative and manic symptoms (as measured by the CDSS and YMRS scales) during and immediately after the infusions.

Segmiller et al. [34] reported a case series consisting of six TRD patients who received six ketamine infusions (0.25 mg/kg over 40 min). One patient decided to discontinue before receiving all infusions due to the transient dissociative effects, but there were no other significant adverse effects.

Szymkowicz et al. [35] reported three patient case reports of repeated IV ketamine administrations (0.5 mg/kg over 40 min) according to individualized needs. One patient received 16 ketamine infusions over the course of a year. The patient did not have any adverse effects and returned to full functionality in all aspects of life. The second patient, with a significant history of suicide attempts and hospitalizations, received a total of 34 ketamine treatments over 12 months, and the only adverse effects reported were an exacerbation of pre-existing cognitive difficulties (driving and forgetting to take the exit, word-finding difficulties, concentration difficulties) and insomnia. These adverse effects were concurrent with a reduction in the antidepressant efficacy of the infusions (tolerance), beginning with the 20th treatment. A third patient, diagnosed with Bipolar II and “cluster-c traits”, received a total of 32 infusions over 12 months, and experienced two transient hypomanic states during that time.

Blier et al. [36] reported on a case of a severely depressed women who experienced a tremendous improvement in her depression and cognitive function, without any adverse effects other than a transient metallic taste and transient derealization, despite receiving a total of 59 IV ketamine infusions (the majority 0.5 mg/kg over 40 min) over five months.

Lopez-Diaz et al. [37] reported a single case of a patient with Bipolar TRD who received six ketamine infusions (0.5 mg/kg over 40 min) over 2 weeks. The authors reported that the only adverse effect the patient experienced was moderate transient sedation during and immediately after the infusions.

The publications in our review described 333 TRD subjects who received six or more parenteral ketamine treatments, 8 of whom (2.4%) discontinued treatment due to ketamine-related adverse effects.

## 3. Discussion

Consistent with a recent survey aimed at characterizing providers of ketamine for depression [15], most of the providers of parenteral ketamine in our survey were psychiatrists, and based in a private practice setting. It should be noted, however, that there was a substantial number of non-psychiatric providers who administered parenteral ketamine for the treatment of major depression or bipolar depression. The number of ketamine providers at the time of the survey’s administration was estimated to be between 50 and 100 [8], which indicates that our sample of 27 included responders represented a substantial percentage of ketamine providers (27‒54%).

According to their self-reported data, respondents to this survey cumulatively had treated 6630 patients with parenteral ketamine at the time of completing the survey (median = 80 patients), and 72% of those patients had received more than five parenteral ketamine administrations.

The respondents in our survey reported that less than 1% of the patients they treated with parenteral ketamine required discontinuation due to an adverse effect. The most commonly reported adverse effect that required discontinuation was psychological distress, highlighting the need for providers and patients to be prepared for the psychoactive properties of ketamine. Other adverse effects related with long-term ketamine treatment, such as symptoms of bladder dysfunction, psychosis, mania and cognitive deficits, were reported in only a small number of cases. Given ketamine’s known propensity to increase blood pressure transiently, it is likely that patients with uncontrolled hypertension were less likely to be treated by the providers, and thus extreme caution should be exercised regarding treating patients with uncontrolled hypertension.

This survey study is the first, to our knowledge, to characterize the long-term adverse effects of parenteral ketamine for depression in a real-world cohort across multiple providers. However, this study does have several limitations. First, as with most survey research, the responses may be subject to recall bias. It is also possible that respondents to our survey may have been motivated to under-report adverse effects. However, evidence from the surveys does not support this, and suggests a proclivity by respondents to over-report, seeing as many adverse events that were reported by respondents did not meet the criteria stated in the survey questions and had to be eliminated. For example, nine cases of addiction-like behavior were reported altogether, but the details provided prompted all of them to be excluded from the descriptive analysis. These details included patients having pre-existing substance abuse at the time of starting ketamine treatments, requests for higher doses of ketamine due to incomplete improvement of depression without any other abuse-like behaviors, cases where ketamine was being administered for non-depression indications such as pain (often at higher doses than used for depression), and cases where abuse behavior was manifested toward intranasal ketamine prescribed for home use between parenteral treatments. In order to not discourage the completion and return of the survey, the survey did not query providers for details of AE surveillance or treatment protocols (e.g., dose, infusion rate, etc.), and thus possible differences in AE rates due to differences in those procedures could not be determined.

Our finding of a low adverse event rate with repeated administrations of parenteral ketamine are consistent with the small but growing number of published reports describing multiple IV ketamine infusions to treat depression, identified in our literature review. In those reports, repeated (six or more) ketamine treatments were reported for 333 subjects with TRD, and there was a reported 2.4% discontinuation rate (*N* = 8) due to adverse effects. While this is approximately three times the rate reported in our survey, it is nevertheless still very low. The modestly higher rates in the published reports may be a result of the inflexibility of the dosing regimens.

Ketamine is a controlled substance (Schedule III), and illicit use has been well documented. Users who misuse or abuse ketamine have reported having cravings, physiological tolerance and possibly withdrawal symptoms upon ketamine cessation [9]. It is notable that we did not identify a single case of reported addiction attributable to parenteral ketamine administration for treating depression, in either our survey or the literature review, given that this one of the most commonly expressed concerns regarding repeated ketamine injections for treating TRD [8,9,38]. This may be because parenteral ketamine treatments are generally conducted in a controlled, monitored environment. Abuse may be more reported than it is with other routes of administration, such as intranasal, which allow patients to self-administer ketamine outside of a professionally monitored setting. Notably, a high-profile published case of repeated ketamine administration for the treatment of TRD, which resulted in apparent tolerance and addiction, involved intranasal ketamine prescribed to a patient for self-administration [39]. In our survey, the only respondent reports of emergent addiction behavior in patients receiving parenteral ketamine for TRD involved patients who were concurrently prescribed intranasal ketamine to self-administer between parenteral administrations (*n* = 3), and the addiction behavior was expressed in the use of the intranasal formulations. While we did not have the ability to assess the possible impact of concomitant medications on incidences of adverse effects, it is wise to be aware of drugs that may potentiate the likelihood of adverse effects, such as drugs that are strong inhibitors of the cytochrome p450 enzymes, CYP2B6 and CYP3A4, which play important roles in metabolizing ketamine.

It is noteworthy that a survey study of South Korean providers of repeated ketamine infusions for chronic pain reported the average dose administered was 1 mg/kg, but produced few serious adverse events, and the treatment was perceived by providers as very safe and tolerable [16].

Furthermore, also consistent with the general safety of parenteral ketamine, Riva-Posse et al. [40] reviewed a total of 684 ketamine infusions (0.5 mg/kg over 40 min) administered to 66 patients in the Department of Psychiatry at Emory University, between 2014 and 2016, for changes in blood pressure during and immediately after the infusions. Measurements were taken every 10 min and they found only modest and transient increases in blood pressure, and no incidence in which treatment had to be discontinued due to blood pressure changes.

## 4. Methods

### 4.1. Survey

An electronic survey, developed by the study authors, was distributed to ketamine providers between November 2017 and January 2018. Providers who administered ketamine parenterally were identified using two internet directories from the following websites: (1) The KRIYA Ketamine Research Institute [41], and the (2) Ketamine Advocacy Network [42].

The KRIYA Ketamine Research Institute is an organization “devoted to understanding the therapeutic properties of ketamine (and related medicines)”, whose self-proclaimed mission is “to bring together the most rigorous science and an appreciation of spirit in the study of psychospiritual medicines such as ketamine.” The Ketamine Advocacy Network’s mission is to “spread awareness of ketamine therapy for treatment-resistant depression, bipolar and PTSD and to make this treatment available and affordable for all who need it.”

The two websites’ directories were used to target providers for our study because they both included the routes of administration that each listed provider utilized to administer ketamine. There were 69 unique providers, with contact information, who indicated they utilize parenteral ketamine, listed on the KRIYA and Ketamine Advocacy Network website directories.

The survey, with a cover letter, was distributed to those 69 identified providers of parenteral ketamine. This survey study was reviewed by the University of California San Diego Human Research Protections Program (HRPP), and deemed to be exempt.

Recipients of the survey were asked to complete the survey if they utilized parenteral ketamine for depression. The survey queried responders about their practice setting and specialty. They were also asked to provide the number of patients they have treated with parenteral ketamine and the number of patients they have treated with various ranges of parenteral ketamine administrations (i.e., 1–5, 6–10, 11–20 and >20). The survey asked providers about serious adverse effects that resulted in the discontinuation of parenteral ketamine treatments. Responders were asked to indicate the number of times treatment was discontinued due specific adverse effects produced by the ketamine treatments, such as bladder symptoms, addiction behavior, psychotic symptoms (persisting after ketamine administration), psychological distress during treatment, cognitive deficits, mania/hypomania and hypertension. Responders were also asked to list all “other” adverse events they witnessed among patients they treated with parenteral ketamine that necessitated discontinuation. Respondents were asked to provide details about each serious adverse event they reported via a series of questions related to the clinical presentation, the outcome, and the dose of ketamine. They were also asked to rate the overall safety of ketamine compared to conventional antidepressants using a Likert scale (i.e., much safer, slightly safer, about the same, slightly less safe, and much less safe).

Responses were collected using an online survey tool, Survey Monkey^®^ [43]. Respondents who did not at least complete questions about the number of treatments they have provided, and the numbers of serious adverse events, were eliminated from the analysis.

The information about the adverse events provided by the providers was reviewed, and any adverse event that did not conform to the stated criteria (e.g., non-parenteral route, non-depression indication for treatment, etc.) were not included in the analysis. The qualifying events were characterized using descriptive statistics.

### 4.2. Literature Review

PubMed searches of publications published between 2000 and 2019 were conducted using the key words “repeated ketamine”, alone and in combination with each of the following words separately: “intravenous,” “intramuscular” and “depression.” Review papers were eliminated, as were papers that were a duplicate report of a previous cohort (additional analysis). Returned results were reviewed to determine if they met the criteria of describing results of the administration of six or more parenteral injections of ketamine for the treatment of depression in humans with a diagnosis of major depressive disorder (MDD) or Bipolar disorder. The papers meeting these criteria were reviewed in depth, extracting information such as the dosing regimen, the number and frequency of ketamine administrations, and the reported adverse events.

## 5. Conclusions

The results of this survey and literature review suggest that the relative incidence of significant adverse effects produced by repeated parenteral ketamine administration for depression in a monitored setting is low, and that repeated parenteral ketamine represents a reasonably safe treatment modality. However, the frequency of transient blood pressure elevation and dissociative experiences, which can occasionally be highly disturbing, indicate the need for this treatment to be administered in a medically supervised setting.

## Figures and Tables

**Table 1:** Demographics of Survey Respondents.

**Specialty**	***N* (%)**
Psychiatry	14 (51.9%)
Anesthesia	7 (25.9%)
Emergency Medicine	4 (14.8%)
Primary Care	1 (3.7%)
Critical Care	1 (3.7%)
Pain and Palliative Care	0 (0%)
**Practice Setting**	***N* (%)**
Private Practice	25(92.6%)
Academic Medical Center	0 (0%)
Non-Academic Medical Center	2 (7.4%)

**Table 2:** Serious adverse effects reported by providers.

Adverse Effect	Cases Requiring Discontinuation * *N* (%)
Bladder Dysfunction	3 (0.06%)
Addiction **	0 (NA)
Psychotic Symptoms	1 (2%)
Cognitive Deficits	0 (NA)
Psychological Distress	33 (0.5%)
Hypomania	2 (0.03%)
Hypertension	0 (NA)
Nausea ***	6 (0.09%)
Respiratory Distress ***	1 (0.02%)
Seizure ***	1(0.02%)
Total	47 (0.7%)

* Rate of Occurrence = Number of cases/total patients treated (6630), ** 9 cases listed but detailed information revealed none qualified, *** Disclosed in “any other adverse effects” question.

**Table 3:** Adverse events (AE) reported in published papers describing treatment of depression with six or more repeated infusions of ketamine.

Report	Number of Patients	Ketamine Dose and Rate	Route	Dis-Continuation Due to AE	Other Notable AEs	Notes
Aan het Rot et al. [19]	10	0.5 mg/kg /40 min	IV	0	0	No significant AEs
Murrough et al. [20]	24	0.5 mg/kg/40 min	IV	0	0	No significant AEs
Zhou et al. [21]	84	0.5 mg/kg/40 min		1 (1.1%)	0	One patient withdrew due to a manic switch
Archer et al. [22]	11	0.5 mg/kg/40 min	IV	0	0	No significant AEs
Wilkinson et al. [23]	14	0.5 mg/kg/40 min	IV	0	0	No significant AEs
Phillips et al. [24]	41	0.5 mg/kg/40 min	IV	0	0	No serious AE and no evidence of craving or drug-seeking behavior during the infusions or follow-up
Vande Voort et al. [25]	12	0.5 mg/kg/40 min	IV	0	2 (1.7%)	1 subject developed behavioral outbursts and suicide threats during follow-up while hospitalized, and another died by suicide several weeks after the end of follow-up. Authors did not attribute events these AEs to ketamine treatments
Ionescu et al. [26]	14	0.5 mg/kg/40 min for first 3 infusions; 75 mg/kg/40 min for last three	IV	Not reported	Not reported	AEs not reported
Ionescu et al. [27]	26	0.5 mg/kg/45 min	IV	0	0	
Singh et al. [28]	21	0.5 mg/kg/40 min	IV	0	0	15–28% (twice weekly infusions) 6–41% (thrice weekly infusions) All symptoms resolved within 3 h of the infusions
Shiroma et al. [29]	12	0.5 mg/kg/40 min	IV	0	2 (1.7%)	Single episode of vomiting effectively countered by IV labetalol and ondansetron
Diamond et al. [30]	28	0.5 mg/kg/40 min	IV	2 (7.1%)	5 (17.9%)	Two patients discontinued due to adverse reactions
Bryant et al. [31]	6	0.5 mg/kg/40 min	IV	0	5 (83.3%)	panic attack (*n* = 1), rapid cycling of mood with a mild hypomanic episode (*n* = 1), emesis (*n* = 2) during treatment, symptomatic cystitis (*n* = 1), and a hypnogogic hallucination (*n* = 1)
George et al. [32]	10	0.3–0.5 mg/kg	SC	0	2 (20%)	One patient reported the urge to urinate slightly more often. One patient had elevated aspartate aminotransferase (42–60) and alanine aminotransferase (25–44) after 12 treatments of 0.5 mg/kg
Albott et al. [33]	15	0.5 mg/kg/40 min	IV	0	0	No significant AEs
Segmiller et al. [34]	6	0.25 mg/kg/40 min	IV	1 (16.7%)	1 (16.7%)	One patient decided to discontinue before receiving all infusions due to the transient dissociative effects
Szymkowicz et al. [35]	3	0.5 mg/kg/40 min	IV	0	2 (66.7%)	Transient hypomanic states over 12 months
Blier et al. [36]	1	0.5 mg/kg/40 min for most infusions	IV	0	0	No significant AEs
Lopez-Diaz et al. [37]	1	0.5 mg/kg/40 min	IV	0	1 (100%)	Transient sedation during infusion
TOTAL	339			4 (1.2%)	20 (5.9%)	Combined 24 (7.1%)
